# Antimicrobial peptides´ immune modulation role in intracellular bacterial infection

**DOI:** 10.3389/fimmu.2023.1119574

**Published:** 2023-03-28

**Authors:** Diana Ivonne Duarte-Mata, Mario César Salinas-Carmona

**Affiliations:** Department of Immunology, School of Medicine and Dr. Jose Eleuterio Gonzalez University Hospital, Universidad Autónoma de Nuevo León, Monterrey, Mexico

**Keywords:** host defense peptides, immunomodulation, antimicrobial peptides, intracellular bacterial infection, cell-penetrating peptides

## Abstract

Intracellular bacteria cause a wide range of diseases, and their intracellular lifestyle makes infections difficult to resolve. Furthermore, standard therapy antibiotics are often unable to eliminate the infection because they have poor cellular uptake and do not reach the concentrations needed to kill bacteria. In this context, antimicrobial peptides (AMPs) are a promising therapeutic approach. AMPs are short cationic peptides. They are essential components of the innate immune response and important candidates for therapy due to their bactericidal properties and ability to modulate host immune responses. AMPs control infections through their diverse immunomodulatory effects stimulating and/or boosting immune responses. This review focuses on AMPs described to treat intracellular bacterial infections and the known immune mechanisms they influence.

## Introduction

Intracellular bacterial infections are difficult to detect and treat. These bacteria have unique mechanisms to live and replicate in host cells, ensuring their survival and permanence. Therefore, bacteria such as *Mycobacterium tuberculosis*, *Shigella flexneri*, and *Salmonella typhimurium*, among others, contribute to the morbidity and mortality associated with infectious diseases worldwide (1).

Control of these infections is an important task. Although various antibiotics are used to treat intracellular pathogens, there is also increased resistance and the appearance of multiresistant clinical isolates that reduce or eliminate the efficacy and success of these antibiotics (2). It is also important to note that many commonly used antibiotics (β-lactams and aminoglycosides) are poorly permeable and do not reach minimum inhibitory concentrations within infected host cells resulting in low antimicrobial activity and making intracellular bacterial infections even more difficult to eradicate (3).

In this scenario, new and better therapies to treat intracellular infections are urgently needed. Promising alternatives are antimicrobial peptides (AMPs), which control a wide range of infections by their direct bactericidal capacity and/or by modulating the host’s immune response.

Antimicrobial peptides were discovered in the 1980s. They are essential components of the innate immune defenses of multicellular organisms, including humans, animals, and plants. Most of these peptides are small (12 to 50 amino acids), cationic, and characterized by hydrophobic and hydrophilic domains. They can be found in different cell types expressed constitutively or induced in response to infectious and/or inflammatory stimuli (4, 5).

It is worth mentioning that the first investigations of AMPs focused on elucidating their microbicidal activity; however, in the last two decades, it has been shown that these peptides can modulate the innate and adaptive immune response to protect the host against infections. This ability, demonstrated by several authors, has been proven a useful therapeutic strategy in preventing and treating infections by intracellular pathogens (6–8).

This review focuses on how modulation of the immune response by AMPs influences the prevention and treatment of some intracellular bacterial infections.

## Antimicrobial peptides

Antimicrobial peptides (AMPs) are important effector molecules in the innate immune response that were originally studied for their direct antimicrobial activities. These molecules have evolved to provide a broad range of protection against various pathogenic microbes, including Gram-positive and Gram-negative bacteria, viruses, archaea, fungi, and parasites. It is important to highlight that these peptides are active against microbes resistant to conventional antibiotics (9–11). AMPs are a widely distributed family in various life forms, from microorganisms to humans (12). Research has identified or predicted more than 3500 cationic peptides from six life kingdoms (9, 11).

AMPs have tremendous variability and diversity in structure and sequence; however, most of these peptides share common features, such as being typically shorter than 50 amino acids, having a cationic net charge provided by Arg and Lys residues, and an amphipathic structure. Currently, there are various ways to classify AMPs. It can be according to their origin, function, or structure; usually, most peptides can be included into four structural categories based on their secondary structure: (i) amphipathic α-helical peptides that include the cathelicidin LL-37, (ii) β-strands stabilized with a variable number of disulfide bonds in which defensins are included, (iii) peptides enriched in one or two amino acids, including tryptophan-rich peptides such as indolicidin, and (iv) loop peptides with one disulfide bridge such as bactenecin (10, 13, 14).

However, the most studied AMP families are the mammalian defensins and cathelicidins. Defensins are 2- to 6-kDa peptides with six conserved cysteine residues and three disulfide bridges (10). Based on the arrangement of these residues, they are classified into α-, β-, and θ-defensins. Humans express six α-defensins in granules of neutrophils and Paneth cells termed Human Neutrophil Peptides (HNP) or are secreted by monocytes, macrophages, mast cells, and natural killer (NK) cells (13). Moreover, human β-defensins (hBD) are normally expressed by epithelial cells of the integument and mucous membranes. Their expression can be constitutive or induced by stimulation with TLR ligands or certain types of cytokines, such as tumor necrosis factor (TNF) or interleukin-1β (15). θ-defensins are macrocyclic peptides that are not produced in humans (16). This type of defensin has only been found in different monkey and orangutan species; in rhesus monkeys, its expression has been detected in neutrophils and monocytes (17).

On the other hand, cathelicidins consist of a highly conserved 14 kDa N-terminal cathelin-like pro-domain, followed by a signal peptide and a C-terminal ‘mature’ peptide region. In humans, there is only one cathelicidin gene (CAMP) expressed as an 18 kDa precursor pro-protein called hCAP18, which is subsequently cleaved to the well-characterized cationic peptide LL-37, which is expressed constitutively or induced by the presence of infections and/or inflammatory stimuli in immune and structural cells (9), highlighting an important role of 1,25-dihydroxy vitamin D_3_ in the induction of this peptide (18). Furthermore, it has been shown that this vitamin increases pattern recognition receptors (PRRs), such as TLR2 and CD14 (19, 20); therefore, it represents another mechanism by which cathelicidin transcription is indirectly increased.

Both defensins and cathelicidins have rapid, potent, and direct antimicrobial activity against several types of bacteria. The efficacy of AMPs against specific types of bacteria will depend on the tissue where the peptide is commonly expressed. For example, β-defensins in the integument show activity against *Staphylococcus aureus* and *Pseudomonas aeruginosa*, two important pathogens responsible for skin infections. Likewise, the mechanisms of action are varied and usually depend on the peptide itself and the pathogen to be eliminated.

Several AMPs kill microbial pathogens by disrupting bacterial membranes since the cationic charge of the peptides attracts them to negatively charged microbial membranes. Also, their hydrophobic properties enable them to insert and disrupt membranes, subsequently leading to leakage of bacterial cell contents and death (21). This rather simplified scenario of AMPs´ selectivity toward bacteria is due to the presence of anionic lipids, lipopolysaccharides (Gram-negative bacteria), or teichoic acids (Gram-positive bacteria) that confer a far more negative charge than mammalian cell surface membranes. This membrane interaction is a key factor for the antimicrobial activity of AMPs, both when the membrane is targeted and when an intracellular target must be reached (12, 22–24). Other mechanisms of action can act indirectly by translocating across the membrane of bacteria targeting intracellular molecules such as nucleic acids or proteins, therefore affecting processes like transcription, replication, or protein synthesis (25–27).

Besides the broad antibacterial activity, more recent studies have indicated that AMPs have the ability to link innate and adaptive immune systems and modulate the magnitude of the immune response to protect against infection, regulate inflammation, and influence immune homeostasis. The large repertoire of immunomodulatory activities associated with AMPs is very large. It includes leukocyte recruitment, chemotaxis stimulation, pro- and anti-inflammatory cytokine induction, endotoxin neutralization, as well as activation and differentiation of immune cell lines, among others. Among the most studied peptides with this capacity are defensins and cathelicidins from different species (28–31). In the following section, the different ways in which AMPs influence immune responses will be discussed in depth.

## Immune response modulation mediated by AMPs

Antimicrobial peptides have a dual ability. They can provide host protection against a wide range of pathogens, first, through their direct antimicrobial activity and second, through their ability to modify the immune system by modulating innate and adaptative immune responses. The same antimicrobial peptide can have both properties or only one or the other (32–35).

The immunomodulation exerted by several AMPs has been proven in several studies, showing that these peptides have the capacity to affect innate and adaptive immune system responses by suppressing or enhancing the response. This capacity is highly varied and is influenced by various factors such as environmental stimuli, the cells involved, the interaction with different receptors, the signaling pathways involved, and the transcription factors to which they bind (36). It is worth mentioning that many AMPs have been shown to have pleiotropic effects depending on the cell on which they act; likewise, it has been seen that the concentration of these peptides plays an important role in the mechanism of action that is triggered (37).

## Innate immune responses are targets for AMPs

Regarding innate immunity, AMPs, such as α-defensins and cathelicidins, can be found in granules of neutrophils and NK cells. Their main function is the intracellular killing of ingested pathogens; however, these peptides are also released into the extracellular milieu and can act as chemokines since they can recruit different cell types, including neutrophils, eosinophils, mast cells, monocytes, and lymphocytes to the infection site (13, 38). This same chemotactic effect can also be exerted by some synthetic AMPs, which include the so-called Innate Defense Regulator Peptides (IDR peptides) that have been shown to attract certain cell types, including monocytes and neutrophils (39, 40).

The mechanisms of action used to achieve this end are by inducing the secretion of chemokines such as MCP-1, RANTES, and IL-8, and/or the increased expression of certain receptors, such as CCR2 and CXCR4, as well as membrane-associated G protein-coupled receptors (GPCRs), among which formyl peptide receptors stand out (41, 42). Likewise, chemokines can act similarly to defensins since they have antimicrobial properties demonstrated *in vitro* by eliminating pathogens such as *Listeria monocytogenes* and *E. coli* (43, 44).

In addition to their chemotactic activity, peptides like HNP-1 (Human Neutrophil Peptide) can enhance the elimination of intracellular pathogens by macrophages through phagocytosis of apoptotic neutrophils and peptides (45). Furthermore, HNP1–3 peptides can activate macrophages and enhance their phagocytosis *in vitro* and in murine models *in vivo* (46). In the same way, it has been observed that cathelicidin LL-37 can activate and enhance phagocytosis by neutrophils. This mechanism is mediated by the synthesis of the chemokine CXCL8 promoted by mitogen-activated protein kinase (MAPK) p38 and extracellular signal-regulated kinase (ERK) (47). On the other hand, it has been seen that LL-37 can inhibit neutrophil apoptosis and, thus, prolong the life span of this cell line by activating formyl-peptide receptor-like 1 (FPRL1) and the P2X7 nucleotide receptor (48). Additionally, the immune modulation exerted by some AMPs includes the capacity to induce reactive oxygen species production and facilitate the formation of neutrophil extracellular traps (49, 50).

In addition to influencing certain neutrophil processes, some AMPs can affect other cell types. Such is the case of cathelicidin LL-37, which has been seen to increase the ability of mast cells to detect pathogens by increasing the expression of TLR4 in LAD2 mast cells, which could be regulated by GPCRs (51). It has also been shown that this same peptide increases the differentiation of monocytes to macrophages with a proinflammatory phenotype, and, in addition, it can decrease the secretion of Interleukin-10 (IL-10) and increase that of Interleukin-12 (IL-12) in macrophages with an anti-inflammatory phenotype (52).

At the same time, certain anti-inflammatory effects have been observed by AMPs. Therefore, it could be deduced that these peptides have an important role in regulating inflammatory processes. Several studies highlight the importance of the anti-inflammatory effects of AMPs since it has been shown that there is a severe inflammatory phenotype in cathelicidin-deficient mice compared to wild-type mice (53). It has also been seen that adding certain peptides can help control the inflammation present in different diseases. Such is the case of the peptide IDR-1002, which reduces the inflammation present in infection and alveolar macrophage infiltration when applied *in vivo* (54).

The immune modulation exerted by AMPs includes the capacity to modulate cytokine production. In this regard, it is important to add that AMPs can promote inflammatory and anti-inflammatory cytokine production. The same peptide can mediate this effect at the same time. HNP1-3 increases the production of TNF-α, IFN-γ, and IL-1 while decreasing the production of IL-10 by monocytes. Likewise, *in vivo* support of their promotion of inflammatory responses, with intratracheal instillation of HNP1-3, enhances the production of TNF-α, MCP-1, and MIP-2, a good example of this property (55). It has been proposed that the downregulation of certain inflammatory mediators is due to the interaction of AMPs with TLRs since it has been scientifically demonstrated that peptides, such as LL-37, can inhibit the secretion of proinflammatory cytokines in monocytes stimulated with TLR2, TLR4, and TLR9 agonists. Likewise, when this cathelicidin is added to peripheral blood mononuclear cells stimulated with LPS, an inhibition of the production of the cytokines TNF, IL-1β, and IL-6 is observed (56). On the other hand, LL-37 can also increase TLR3-mediated responses and thus promote IL-1B secretion (57); similarly, combining this peptide with TLR3, TLR2, and TLR5 agonists increase the production of IL-6 and CXCL8 (58).

Other mechanisms by which AMPs can modulate the innate immune response include the capacity to act as opsonins, activate or suppress the activation of the classical pathway of the complement system, and activation of the NLRP3 inflammasome (59, 60).

## Adaptive immune responses modified by AMPs

AMPs can also affect the responses of the adaptative system. In this aspect, antimicrobial peptides are excellent molecules that serve as a link between innate and acquired immune responses since it is known that different types of defensins and the human cathelicidin LL-37 help initiate the adaptive antimicrobial immune responses because they recruit antigen-presenting cells (APCs) at infection sites, including immature dendritic cells (iDCs), B lymphocytes and macrophages (61). The finding that HNP1-3 and the human β-defensins (hBD) 1-3 are selectively chemoattractant for human iDCs by interacting with CCR6 support this fact (62–64). Besides recruitment, AMPs also have the ability to activate and promote dendritic cells (DCs) maturation since it has been shown that mouse β-defensin (mBD2) 2 upregulates the expression of costimulatory receptors, such as CD40, CD80, and MHC II, similar to the effects seen with hBD1, hBD3, and HNP-1. This expression is presumed mediated by interaction with TLRs 1 and 2, resulting in MyD88 signaling (65). Likewise, when exposed to LL-37, immature monocyte-derived dendritic cells displayed a significant increase in endocytic capacity (66), increasing the ability to activate naïve T cells. This noteworthy activity is important in the induction of Ag-specific immune responses since DCs are the principal cells responsible for this specific response (55).

It is well known that AMPs can influence T-lymphocyte polarization. This effect was demonstrated in various studies where DCs activated in the presence of peptides, such as LL-37 and mBD2, causing the induction of a Th1-type response. This same effect is also observed in Langerhans cells, which are activated by hBD3, inducing IFN-γ secretion by T lymphocytes, suggesting bias towards Th1 responses (66–68).

Besides the chemoattraction of DCs, defensins, such as hBD1 and hBD3, possess the ability to chemoattract different lymphocyte subtypes, including naïve and memory CD4+ and CD8+ T cells favoring, even more, the effector mechanisms of adaptative responses (16).

In the context of humoral immune responses, defensins can also modulate this response; however, they do it differently since it has been demonstrated that they can act as adjuvants and enhance the production of specific IgG antibodies. On the other hand, peptides, such as LL-37, cause a decrease in the production of the anti-inflammatory cytokine IL-10 by B lymphocytes (69, 70).

Due to all the effects that AMPs can exert on innate and adaptative immune responses, it is thought that the main role of antimicrobial peptides in bacterial clearance is to modulate the immune response rather than direct the elimination of pathogens. This idea comes from the fact that many antimicrobial peptides lose their antimicrobial activity under physiological conditions. In contrast, their immunomodulatory activities remain present and can be observed in the addition of serum and when tested in animal models (71–73). A novel cathelicidin from the tree frog, PopuCATH, lacks direct antibacterial activity *in vitro*; however, its intraperitoneal injection in mice before bacterial inoculation significantly reduced the bacterial burden and the inflammatory response caused by bacteria (74). Several reports demonstrate that immunomodulation is the key to promoting pathogen clearance. It has been proposed that the *in vivo* antibacterial activity of some AMPs is mediated primarily through their immunomodulatory effects rather than direct bacterial killing (12).

There is also evidence that some AMPs provide protection when applied topically or systemically through intravenous, intraperitoneal, and subcutaneous routes and are effective when delivered prophylactically or after inoculation with bacteria (4).

Taking all these immune properties together, we can say that the promotion of pathogen clearance by antimicrobial peptides is probably due to their multiple immunomodulatory effects on the host response and the combination of different immunological pathways affected by AMP modulation since the increase in bacterial phagocytosis by macrophages until the promotion of dendritic cell maturation, indicates that the main role of peptides in infection resolution is by acting on the host’s immune response (46, 66).

## AMPs against intracellular bacteria

Generally, bacteria are divided into two groups, extracellular and intracellular. Extracellular bacteria live outside the cells of the infected host and in environmental niches. Intracellular bacteria enter a host cell and replicate, causing infection. Intracellular bacteria possess several strategies to survive inside cells; therefore, they can protect themselves from the components of the immune defenses, such as antibodies and complement (6, 75). Obligate intracellular bacteria are part of the intracellular bacteria group. These pathogens fully depend on the metabolism of the host´s infected cells and cannot survive outside these cells. Among these are *Chlamydia* spp., *Rickettsia* spp., and *Coxiella burnetii*, to mention a few. Facultative intracellular bacteria also exist, and unlike obligate bacteria, they can survive and replicate inside and outside host cells. This group includes *Salmonella* spp., *Francisella tularensis*, *Brucella abortus*, *Nocardia brasiliensis*, *Listeria monocytogenes*, and *Mycobacterium tuberculosis* (76).

Intracellular bacterial infections are difficult to treat because, unlike extracellular bacteria, the antibiotics used for the infection must have the ability to penetrate the plasma membrane of the host cell to eliminate them. In addition, it must do so without harming the infected cell. Many AMPs possess this remarkable activity against intracellular bacteria; however, these peptides have been reported to a lesser extent (6).

*In vitro* and *in vivo* experiments demonstrate the direct antibacterial activity on some intracellular bacteria, such as *Nocardia* spp., *Mycobacterium tuberculosis, Listeria monocytogenes*, including resistant *Salmonella enterica* (**Table 1**) (77–82). However, the ability of these peptides to penetrate the host cell was not evaluated, reflecting the physiological conditions where the bacterium is normally found. For this reason, these peptides must be able to enter the host cell to perform their function.

**Table 1:** Antimicrobial activity of AMPs against intracellular bacteria.

Peptide	Bacteria	Mechanisms of action	Dose-Response Relationships	Experimental phase (model)	Reference
hBD-3	*Nocardia farcinica*	Exhibits direct bactericidal activity in pure culture	LD_90_ 16 μg/ml	*In vitro*	75
	*Nocardia nova*	Exhibits direct bactericidal activity in pure culture	LD_90_ 16 μg/ml	*In vitro*	75
LL-37	*Nocardia farcinica*	Exhibits direct bactericidal activity in pure culture	LD_90_ 32 μg/ml	*In vitro*	75
	*Nocardia nova*	Exhibits direct bactericidal activity in pure culture	LD_90_ 32 μg/ml	*In vitro*	75
HNP 1-3	*Nocardia nova*	Exhibits direct bactericidal activity in pure culture	LD_90_ 64 μg/ml	*In vitro*	75
	*Nocardia asteroides*	Exhibits direct bactericidal activity in pure culture	LD_90_ 32 μg/ml	*In vitro*	75
Bovine Indolicidin	*Nocardia nova*	Exhibits direct bactericidal activity in pure culture	LD_90_ 8 μg/ml	*In vitro*	75
	*Nocardia asteroides*	Exhibits direct bactericidal activity in pure culture	LD_90_ 64 μg/ml	*In vitro*	75
	*Nocardia brasiliensis*	Exhibits direct bactericidal activity in pure culture	LD_90_ 64 μg/ml	*In vitro*	75
GW-Q6	*Salmonella Choleraesuis*	Inhibits bacterial growth (The peptide has a bacteriostatic, but not bactericidal effect)	MIC MDR strains 4–32 μg/mL MIC WT strains 8–64 μg/mL	*In vitro*	77
HNP-1	*M. tuberculosis*	Exhibits direct bactericidal activity in a pure culture mediated by disruption of the mycobacterial cell wall/membrane and inhibition of DNA biosynthesis.	MIC 2.0 µg/ml	*In vitro*	80
CyLoP-1	*Salmonella typhimurium*	Causes a bacteriostatic effect.	MIC 10 µMol	*In vitro*	95

There are multiple reports that AMPs can be internalized in different cell types. The cell that they can enter will depend on the type of peptide. In addition, esculentin-1a-derived peptides can penetrate the plasma membrane of bronchial cells and promote the rapid clearance of intracellular *Pseudomonas aeruginosa* since there is a ~ 40% and 60% decrease in the number of intracellular bacteria one hour after exposure (83).. Likewise, peptides like N6NH2 and their derivative, GUON6NH2, can be internalized in infected macrophages and exert their antibacterial activity against *Edwardsiella tarda*, thus helping reduce intracellular bacterial numbers (84). Another peptide that was able to enter murine and human macrophages *in vivo* is mature bovine neutrophil β-defensin 4. This peptide showed potent bactericidal activity against *Mycobacterium bovis* and *Mycobacterium tuberculosis* in macrophages, reducing the intracellular survival of mycobacteria (85).

Another mechanism by which peptides can directly kill intracellular bacteria without crossing the host cell membrane is by inducing the synthesis of certain peptides by infected cells. An example would be macrophages. There is scientific evidence that cathelicidin LL-37 is upregulated in macrophages infected with *Mycobacterium* spp., and apart from having direct bactericidal activity, this peptide helps macrophages eliminate the bacteria by facilitating the fusion of mycobacterial phagosomes to lysosomes (7). Moreover, in a murine model of *Salmonella typhimurium* infection, the increased expression of the murine cathelicidin-related antimicrobial peptide (CRAMP) in macrophages is regulated by reactive oxygen intermediates and intracellular proteases, which highlights the cooperation between innate bactericidal mechanisms that mediate impaired cell division of *Salmonella* within macrophages (86).

It is hypothesized that macrophages can endocytose AMPs, such as β-defensins and LL-37, increasing the lysis of intracellular bacteria (7, 85). Furthermore, peptides like lactoferricin B, at the concentration where no bacteriostatic or bactericidal effect is produced, have the ability to reduce the entry of an intracellular pathogen into target cells. This mechanism has been demonstrated with the bacterium *Listeria monocytogenes* (87).

Although several studies currently focus on the antibacterial activity of AMPs, only a minority kill intracellular bacteria. Even fewer peptides can internalize in eukaryotic cells to reach their target objective. Therefore, modifying peptides to improve their bactericidal functions is very common to kill multiresistant strains and reduce their cytotoxic potential. An example of a modified peptide is a cationic antimicrobial peptide (CAMP) coupled with cinnamic acid derivatives, which show an increased ability to inhibit intracellular growth of clinical isolates of *M. tuberculosis in vitro* at low concentrations (88). It has also been seen that specific regions of very large peptides can maintain their antimicrobial activity and lose their off-target effects that can harm the infected organism (89).

In addition to AMP modifications, these peptides can be combined with conventional antibiotics. These combined therapies have the expected synergism, resulting in an improvement in clinical outcomes. Proof of this is that using AMPs containing all-D amino acids or defensins together with antituberculosis drugs (isoniazid and rifampicin) enhances the efficacy of these antibiotics against *Mycobacterium tuberculosis in vitro*, *ex vivo*, and *in vivo* since reductions in the minimum inhibitory concentrations (MICs) of these agents and significant clearance of the bacterial load from the lungs, liver, and spleen of infected animals have been observed. This observed effect is due to AMPs possibly facilitating access of the drugs to intracellular targets (90, 91).

**Figure 1:**
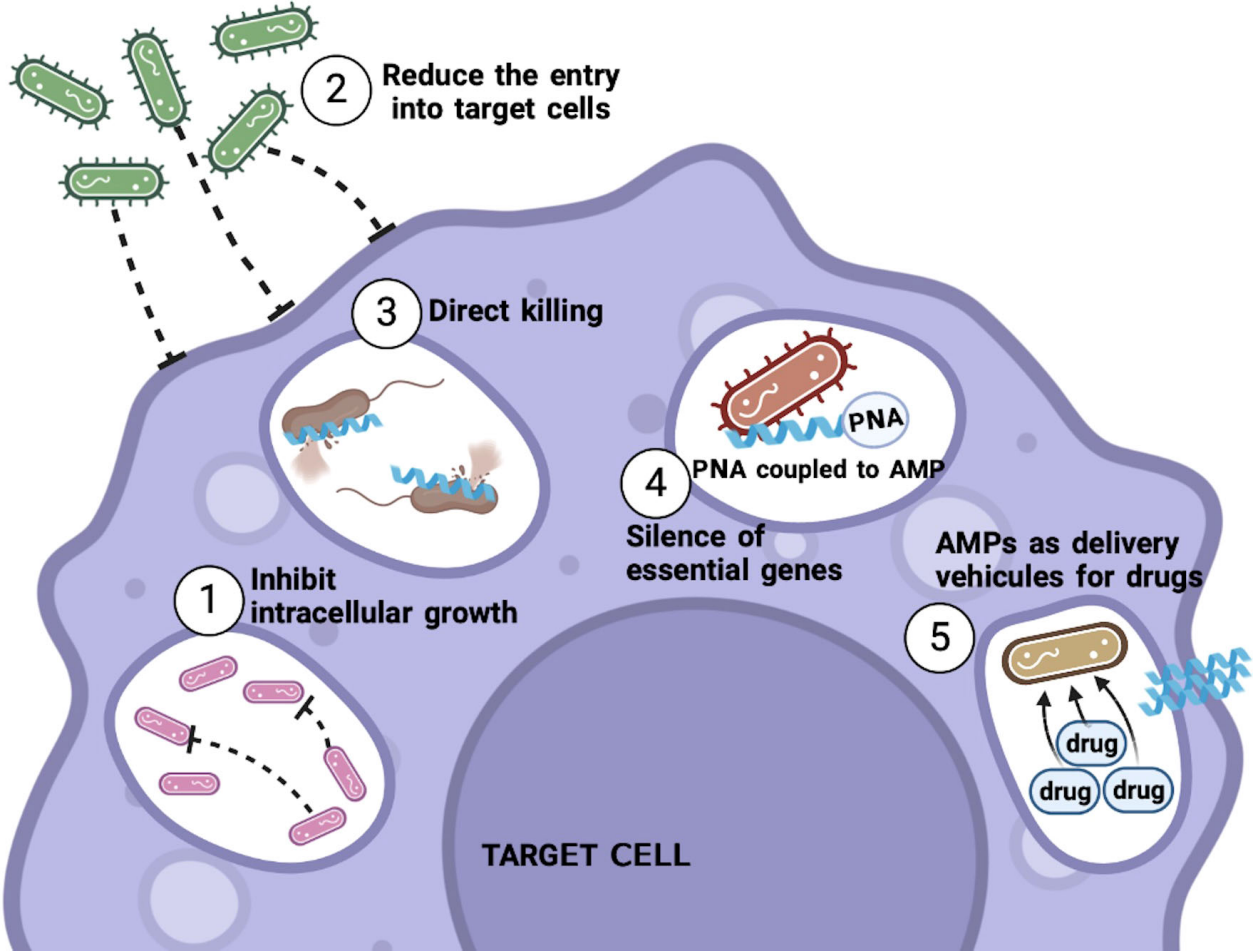
Mechanisms of action of AMPs against intracellular bacteria. AMPs can use different mechanisms to kill intracellular bacteria. Among these are the inhibition of the growth of the bacteria, as well as avoiding or reducing the entrance of the bacteria to the target cell, and the ability to penetrate the infected cell to kill the bacteria that are already inside. Thus, AMPs linked to different molecules can also be used as PNAs, to try to silence essential genes for the bacteria or attached with commonly used antibiotics to allow these drugs to enter the cell and be able to eliminate the bacteria. .

In summary, the mechanism of action of each particular peptide may vary (**Figure 1**) and depend on the physical and chemical characteristics of the peptide in question, as well as the interactions of AMP with both the bacteria and the host cell. However, it is important to remember that the peptide must be able to eliminate the pathogen even when it is within its preferred niche, the cell.

## Cell-penetrating peptides´ antibacterial activity against intracellular pathogens

Cell-penetrating peptides (CPPs) are short peptides with a maximum length of 30 amino acids with a positive net charge. They penetrate the biological membranes of different cell types in a noninvasive manner and drive the internalization of bioactive cargo in cells (92). Most CPPs lack bactericidal activity; however, within this group of peptides, we can also find some that have the characteristic of killing bacteria directly. Also, as mentioned before, like CPP, some AMPs penetrate biological membranes of different cell types, and certain types of bacteria lead to enhanced killing efficacy (93). This dual ability to behave at the same time as a CPP and an AMP, namely, to be able to cross the host cell membrane without destroying its integrity and interrupting the integrity of bacterial membranes for their elimination, is attributed to the difference in electrostatic charges between mammalian and bacterial membranes (94). Thus, although CPPs and AMPs share similarities between their physicochemical properties and, very importantly, their mechanism of action, many researchers treat them as two different groups; however, certain authors question whether they belong to different groups (95).

The main objective of using CPPs is the transport of cargo molecules to the cell’s interior. Among the different molecules that bind these peptides, we find biological products such as proteins, oligonucleotides, nanoparticles, conventional drugs, and AMPs (6). In particular, combinations of CPPs and antibiotics are useful for fighting infections caused by intracellular bacteria. While some of these peptides increase the efficacy of conventional antibiotics, others attack intracellular targets to deliver cargo molecules (93). One advantage of CPPs is that they are highly efficient and safe since they have low cytotoxicity and produce no immunological response (96).

As with AMPs, various CPPs have been shown to have a direct bactericidal effect on different bacterial strains, such as *Salmonella typhi*, and an ability to penetrate cell membranes (97). However, these two abilities, bactericidal and penetration, have been tested separately. Therefore, when it comes to targeting these peptides for eradicating intracellular bacterial infections, these two abilities must be tested simultaneously, i.e., the efficacy of the peptide to enter the host cell to kill the bacteria.

Likewise, many CPPs have been evaluated for the delivery of cargo, such as non-cell permeable antibiotics. Although some possess strong bactericidal activity against extracellular bacteria, their therapeutic efficacy decreases when killing intracellular bacteria since they do not reach high therapeutic levels in intracellular compartments. Such is the case of gentamicin, which was conjugated with various CPPs. These conjugates displayed enhanced bacteria killing within the cell compared to unconjugated gentamicin treatment, drastically reducing the number of intracellular *Shigella* or *Salmonella* organisms in infected cells. Moreover, the lysis and destruction of intracellular bacteria after adding CPP-gentamicin conjugates to infected cells was confirmed (3).

An innovative way to conjugate CPPs is with peptide nucleic acids (PNAs). These conjugations allow the passage of PNA into the infected cell and thus silence certain genes that are essential for the survival of the bacteria. They represent a promising therapeutic approach to eradicate intracellular pathogens harboring inside cells. A recent study showed that constructs of CPPs and PNA that target the RNA polymerase α subunit (rpoA) in the intracellular pathogen *Listeria monocytogenes* reduced the expression of rpoA and consequently, a reduction in the expression of other virulence genes were observed. Therefore, silencing the expression of rpoA leads to rapid and direct bacterial cell death, a finding confirmed *in vitro* and *in vivo* (98).

Another promising approach to conjugate CPPs is with phosphorodiamidate morpholino oligomers (PMOs). PMOs are short single-stranded DNA analogs that bind to complementary sequences of target mRNA to block protein translation through steric blockade. Alone, PMOs are unable to enter bacterial cells effectively. Conjugating PMO with CPP increases the delivery of PMO into the target cell. This conjugation inhibits the growth of intracellular bacteria like *Salmonella enterica* in pure culture and reduces their intracellular viability in macrophages. This finding means that a CPP-PMO conjugate can enter macrophages, apparently cross two bacterial membranes, and effectively deliver the PMO cargo to its target in the bacterial cytoplasm (99).

In the same way, reports of conjugates of antimicrobial peptides and cell-penetrating peptides have shown that the resulting union of these two peptides increases their therapeutic efficacy. In addition, the union of the marine peptide N6 with the cell-penetrating peptide, Tat11, increased the capacity to kill *Salmonellae Typhimurium* in RAW264.7 cells and *in vivo* models. The explanation lies in the fact that CPP helps AMP enter the infected cell. After uptake into the infected cells, AMP reached vacuoles and interacted with the bacteria to eliminate them (100).

On the other hand, the modification of peptides known to have antibacterial activity has also been sought to improve their function and/or reduce their cytotoxicity towards eukaryotic cells. Within this category, we find CPPs with N-terminal hydrophobic modifications. These altered peptides showed superior cell penetration in macrophages, lower toxicity than in the original peptide, and improved bactericidal activity, where *Shigella flexneri* clearance is promoted within macrophages (101).

Examples of AMPs and CPPs are shown in **Table 2**. Together they show high therapeutic efficacy against infections caused by intracellular pathogens. CPPs, when used as cargo molecules, can increase treatment options, either by silencing essential bacterial genes for survival or increasing the therapeutic index of conventional antibiotics and certain AMPs, thanks to their high permeability in mammalian and bacterial cells. In this regard, CPPs represent a promising approach to eliminating intracellular bacterial infections.

**Table 2:** Antimicrobial activity of AMPs against intracellular bacteria within the cell.

Peptide	Bacteria	Mechanisms of action	Dose-Response Relationships	Experimental phase (model)	Reference
mBNBD 4	*M.tuberculosis* *M. smegmatis* *M. bovis*	Exogenous addition of mBNBD4 to Raw 264.7 and THP-1 cells reduced the intracellular survival of Mycobacteria relative to control cells.	Not reported	*In Vitro*	83
Gran1	*M. tuberculosis*	Inhibits growth of extra- and intracellular mycobacteria.	Not reported	*In vitro*	87
D-LAK-120-H	*M. tuberculosis*	Inhibited the intracellular growth of the bacterium and prevented bacteria cell aggregation.	EC_50_ 32.2 ± 5.5 µMol	*In vitro* and *ex vivo*	88
Lactoferricin B	*Listeria monocytogenes*	Ability to reduce the entry into target cells.	MIC 31 µMol	*In vitro*	85
Cin + CAMP3	*M. tuberculosis*	Increased ability to inhibit intracellular growth of clinical isolates.	MIC 38.51 µMol	*In vitro*	86
GUON6NH2	*Edwardsiella tarda*	The peptides are internalized into macrophages and reduce intracellular bacterial numbers.	MIC 8 µg/mL *In vivo* dosis 1 µmol/kg	*In vitro* and *In vivo*	82
PRXR	*Listeria monocytogenes*	Reduced expression of rpoA and other virulence genes. Rapid and direct bacterial death and complete clearance of intracellular Listeria.	MIC 1 µMol	*In vitro* and *In vivo*	96
3+Pip-AcpP PPMO	*Salmonella Typhimurium*	PMO-peptide conjugate inhibited intracellular growth and viability in macrophages and pure culture.	MIC 0.156 µMol	*In vitro*	97
Pentyl-P14	*Shigella flexneri*	Bacterial clearance is promoted within macrophages.	MIC 4 µMol	*In vitro*	99
Tat-gentamicina	*Salmonella Typhimurium*	Display enhanced bacteria killing within the cell compared to unconjugated gentamicin treatment, drastically reducing the number of intracellular Salmonella in infected cells by lysis and destruction.	MIC 1 μg/mL	*In vitro*	3
α2H-gentamicin			MIC 1 μg/mL		
α1H-gentamicin			MIC 2 μg/mL		
Tat-gentamicina	*Shigella flexneri*	Display enhanced bacteria killing within the cell compared to unconjugated gentamicin treatment, drastically reducing the number of intracellular *Shigella* in infected cells by lysis and destruction.	MIC 1 μg/mL	*In vitro*	3
α2H-gentamicin			MIC 2 μg/mL		
α1H-gentamicin			MIC 2 μg/mL		

## Bacterial clearance by AMP immunomodulation

When speaking of immunomodulation, a reference is made to the ability of certain molecules or substances to modulate immune responses, whether innate or acquired, to enhance or suppress their effect mechanisms. This approach has proved useful as a therapeutic strategy in various contexts, including treating certain types of cancer, autoimmune diseases, and infectious diseases, such as those caused by *M. tuberculosis* (31). Regarding infectious diseases, immunomodulation offers certain advantages since this approach does not target the pathogen directly but rather selectively boosts immune responses and modulates inflammatory responses to eliminate the infection (102). This approach is beneficial when eliminating infections caused by antibiotic-resistant or intracellular bacteria, which are difficult to eradicate.

Among the molecules capable of modulating immune responses, we find AMPs, agonists of innate immune components, such as Toll-like receptors (TLRs), and other natural bacterial ligands, such as cyclic nucleotides (31). In this review, we focused on AMPs. What makes AMPs unique is that they are endowed with a set of properties (modulation of the host immune response and direct microbicidal capacity) that make them excellent candidates for controlling infections while limiting an excessive inflammatory response (103, 104).

AMPs are essential for controlling infections *in vivo* since it has been shown that cathelicidin-deficient mice display increased susceptibility to bacterial infections of the skin (105), the intestinal tract (106), the cornea (107), the urinary tract (108), and lung (109), indicating that AMPs have an important role in host defense.

Even though it is already widely demonstrated that peptides modulate a wide range of innate and adaptive immune responses, most experiments have been performed on extracellular bacterial infections. Although scientific evidence is scarce regarding intracellular bacterial infections, certain peptides can be highlighted in eliminating these pathogens. In addition, the antimicrobial peptide JH-3 has excellent antibacterial activity against *Salmonella Typhimurium in vitro*. Furthermore, JH-3 had notable protective effects in mice infected with a lethal dose of this bacteria. This conferred protection is mostly because the peptide can inhibit the apoptosis of infected macrophages, with the bonus that certain inflammatory cytokines such as interleukin 2 (IL-2), interleukin 6 (IL-6), and TNF-α are downregulated. This helps increase the host defense to inhibit bacterial infection (110).

Other peptides that help prevent or diminish *Salmonella typhimurium* infection are cathelicidin LL-37 and human β-defensins 2 and 3 (hBD-2 and hBD-3). These antimicrobial peptides, commonly secreted by different cell types, play an important role in the immune response in the intestine (innate gut defenses), where they are secreted by intestinal epithelium cells. Studies have shown that human cathelicidin prevents *S. typhimurium* internalization and maintains epithelial barrier integrity, achieved through increased synthesis of Toll-like receptor 4 (TLR4) in colonic epithelial cells and induction of the proinflammatory cytokine IL-1β gene in response to the bacteria (111). Conversely, hBD-2 and hBD-3 help reduce certain inflammatory cytokines such as IL-6, IL-8, TNF-α, IL-1α, and IL-1β, while increasing the cytokine anti-inflammatory TGF-β. Thus, the invasive and inflammatory potential of *S. typhimurium* is significantly reduced in the presence of these human defensins; however, we must consider that the ability of hBD-2 and hBD-3 to reduce the inflammatory response in infected cells is also due to their killing activity against *Salmonella* (112). This information demonstrates that cathelicidins and defensins have important immunomodulatory roles in gut defenses beyond bactericidal effects.

As previously mentioned, AMPs can also modulate adaptive immune responses, as is the case of bovine lactoferrin (bLf), where oral administration of this peptide as a treatment in mice lethally infected with *S. typhimurium* reduces mortality and leads to an increase in the total IgA, IgG, and IgM antibody response and specific *Salmonella* antigens generated by the infection. It appears that bLf stimulates humoral responses and reduces the bacterial load in the intestine and systemic levels (113). On the other hand, human lactoferrin (hLf) can modulate the anti-inflammatory response in *Listeria monocytogenes* infection and decrease liver colonization by this intracellular pathogen. In mice infected and treated with hLf, the necrotic foci decreased in number and size, and the bacterial load of *L. monocytogenes* in the liver decreased. Likewise, the presence of macrophages and neutrophils, in which phagocytosis could be increased, was demonstrated since *in vitro* studies report that lactoferrin increases the phagocytic capacity of these cells (114). Furthermore, the expression of certain proinflammatory cytokines, such as IL-1β, TNF-α, and IFN-γ, is diminished (80). However, it is a matter of discussion whether this anti-inflammatory capacity is due to the peptide´s antibacterial activity, the immunomodulatory properties of the peptide in question, or both.

Since there is extensive evidence on the antibacterial and immunomodulatory properties of natural AMPs, their sequences, and structures serve as templates for the design of synthetic AMPs, focusing on improving and increasing immunomodulatory and microbicidal functions and trying to eliminate any cytotoxic effects. Some of the well-known and booming synthetic peptides to date are called innate defense regulator peptides (IDR peptides). These have modest or null bactericidal activity *in vitro* and regulate the host’s innate immune response to enhance immune cell recruitment to the site of infection to eliminate the bacteria instead of attacking them directly (31, 115, 116).

One of the IDR peptides that protect against intracellular bacterial infections *in vivo* is IDR-1, which, applied prophylactically, protects against *Salmonella enterica* serovar Typhimurium infection by decreasing colony-forming units (CFU). This protection is attributed to the selective attraction of monocytes and macrophages to the infection site by IDR-1 since this peptide lacks direct bactericidal activity. In addition, the peptide stimulates several signaling pathways that increase chemokines, such as RANTES and MCP-1, which are probably responsible for the recruitment of monocytes and macrophages (39). Other peptides in the same category that have been shown effective against the intracellular bacterium *M. tuberculosis* are IDR-HH2 and IDR-1018, which, when applied as a treatment to mice infected with both a strain susceptible to antibiotics, H37Rv, and a multidrug-resistant (MDR) isolate, show a reduction in lung inflammation, a decrease in the bacterial load, and an increase in the number of activated macrophages at the infection site, presumably due to administration of the peptide, indicating that the peptide is eliminating the bacteria through its immunoregulatory properties (78).

**Figure 2:**
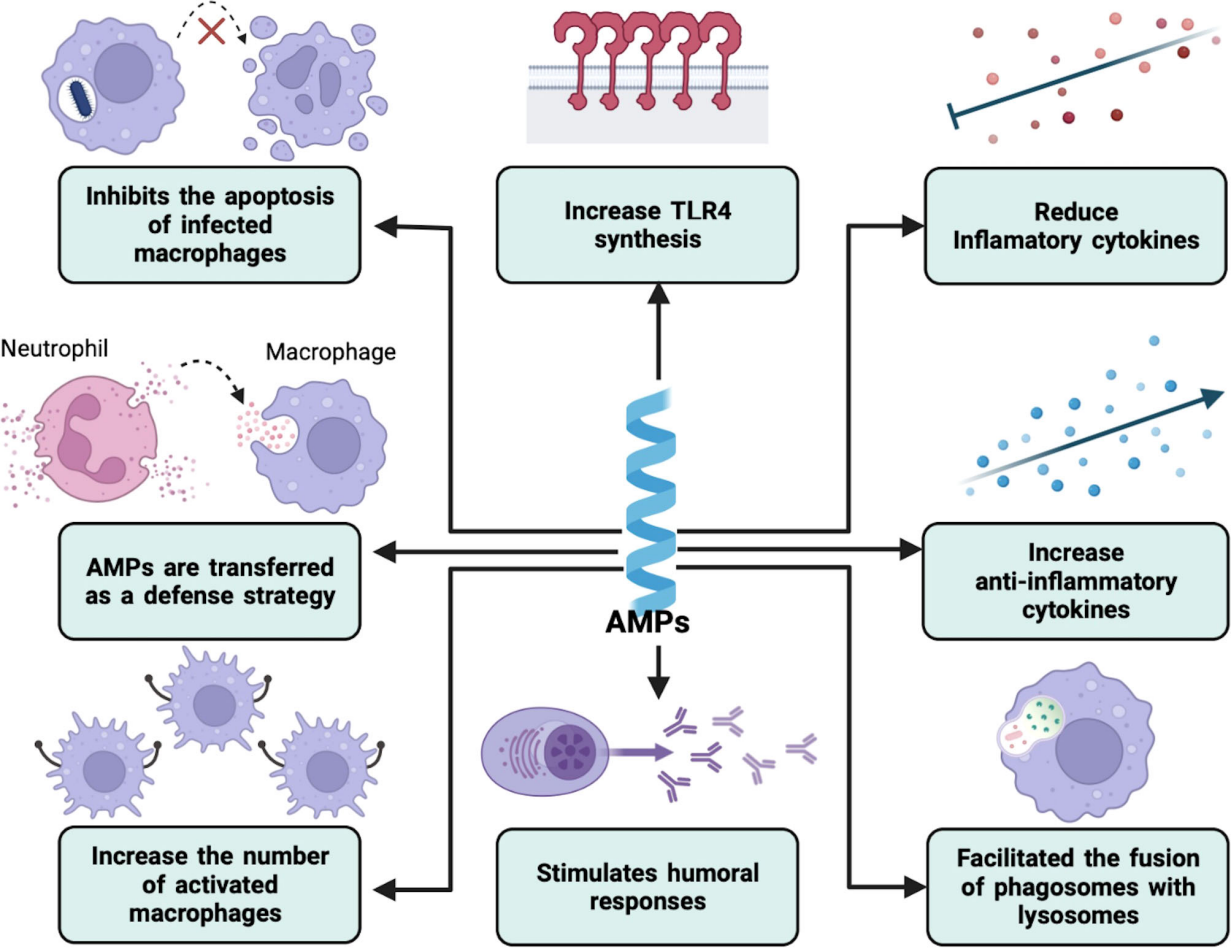
Immunomodulation by AMPs in intracellular bacterial infections. AMPs can modulate the host immune response in different ways. These include the regulation of the secretion of both anti- and proinflammatory cytokines and the expression of certain innate immune receptors. They can also increase the number of active macrophages and facilitate the phagolysosome formation. On the other hand, humoral immunity can also be modified.

**Table 3** and **Figure 2** summarize antimicrobial peptides with immunoregulatory properties.

**Table 3:** Immunomodulation by AMPs in infections by intracellular bacteria.

Peptide	Bacteria	Mechanisms of action	Dose-Response Relationships	Experimental phase (model)	Reference
HNP-1	*M. tuberculosis*	Antimicrobial peptides from neutrophils are transferred to macrophages providing a cooperative defense strategy between innate immune cells.	MIC 2.0 µg/ml	*In vitro*	43 and 80
Beta-Defensin-2 and Beta-Defensin-3	*Salmonella typhimurium*	Helps reduce the inflammatory cytokines IL-6, IL-8, TNF-α, IL-1α, and IL-1β, while increasing the anti-inflammatory cytokine TGF-β, which means that the invasive and inflammatory potential of *S. typhimurium* is significantly reduced.	MIC Not reported	*In vitro*	110
LL-37	*M. tuberculosis* *M. smegmatis* *M. bovis*	Helps macrophages to eliminate the bacteria by facilitating the ability of mycobacterial phagosomes to fuse with lysosomes.	MIC Not reported	*In vitro* and *in vivo*	7
IDR-1018	*M. tuberculosis*	Reduction in the inflammation present in the lung and a decrease in the bacterial load, in addition to an increase in the number of activated macrophages present at the site of infection.	MIC: 16 ± 5.4 µg/mL *In vivo* dosis 32 µg/raton	*In vitro* and *In vivo*	76
Human lactoferrin (hLf)	*Listeria monocytogenes*	Diminish the expression of the proinflammatory cytokines IL-1β, TNF-α, and IFN-γ and decrease bacterial load in the liver.	MIC 1000 µg/mL	*In vitro* and *In vivo*	78
CRAMP	*Salmonella typhimurium*	CRAMP, in conjunction with proteases and reactive oxygen intermediates, impairs *S. typhimurium* replication.	MIC 3.12 µMol	*In vitro* and *In vivo*	79 and 84
JH-3	*Salmonella typhimurium*	Capable of inhibiting the apoptosis of infected macrophages and killing the bacteria within; furthermore, the inflammatory cytokines IL-2, IL-6, and TNF-α are downregulated.	MIC Not reported	*In vitro*	108
LL-37	*Salmonella typhimurium*	Prevents *S. typhimurium* internalization and increases the synthesis of Toll-like receptor 4 (TLR4) in colonic epithelial cells and induction of the proinflammatory cytokine IL-1β gene.	MIC Not reported	*In vitro*	109
Bovine Lactoferrin (bLf)	*Salmonella typhimurium*	Increases the total IgA, IgG, and IgM antibody response as well as that specific to Salmonella antigens generated by the infection and contributes to reducing the bacterial load.	MIC Not reported	*In vitro*	111

## The Potential and challenges of AMPs as an alternative to antibiotics

AMPs represent a promising alternative to be used as anti-infective agents. This fact is largely due to their antibacterial properties, ability to regulate the immune response, and the fact that they are less likely to induce bacterial resistance.

Several recent *in vitro* and *in vivo* studies showed that natural and synthetic AMPs could be used to prevent and eliminate infections. They also showed that the mechanisms of action and the cells involved could vary from one peptide to another. It should be noted that there are currently various preclinical and clinical studies using AMPs; however, it is worth noting that most of the peptides are directed at infections caused by bacteria considered extracellular.

There are no registered clinical studies with peptides directed at intracellular bacteria to date. Among the few preclinical studies considered for these infections is Microcin C7, which has been shown to have excellent activity by inhibiting the growth of *Shigella flexneri in vitro* and *in vivo*, thus representing a therapeutic candidate for gastrointestinal infections (117). Another peptide candidate with the same purpose is Pediocin PA-1, which has been shown effective when administered intragastrically in animal models infected with *Listeria monocytogenes* since it increases the mortality of this pathogen and decreases bacteria translocation to the liver and spleen. It was also found that it does not affect the composition of the intestinal microbiota (118).

There are studies where various compounds are used to promote the induction of specific AMPs, increasing the immune response against infections. These studies have shown favorable results. Adjunctive therapy with vitamin D3 or phenylbutyrate accelerates clinical recovery, where sputum culture conversion and increased expression of cathelicidin LL-37 in immune cells is observed, as well as an increase in macrophage-mediated killing in *ex vivo* experiments (119–121). It is worth mentioning that although there are several *in vitro* and *in vivo* studies demonstrating the potential of certain AMPs to modulate the immune response and help eliminate different types of infections, there are very few clinical studies for this purpose. Among these is the application of human cathelicidin LL-37 to enhance the healing of hard-to-heal venous leg ulcers and the synthetic peptide Mel4 in contact lenses to reduce infections associated with this product (122, 123).

Among the many other clinical studies, we can find peptides targeting respiratory tract infections, skin infections, bacteremia, impetigo, candidiasis, and others. However, this topic is beyond the scope of this review (124).

Unfortunately, many clinical trials using AMPs were terminated for various reasons. One of them is adverse effects. Such is the case of murepavadin peptide in phase III clinical trials, which was found to produce a high number of cases with acute kidney injuries in patients with nosocomial pneumonia (125). Another is that clinical trials show poor efficacy or efficacy that does not exceed the antibiotics in use; one example is the peptide iseganan, which was applied topically in the oropharynx of patients with prolonged mechanical ventilation and did not show a significant reduction in the incidence of ventilator-associated pneumonia (126).

Although it is scientifically proven that AMPs have several desired characteristics in an antibiotic, it is also well known that they often trigger unwanted effects such as cytotoxicity towards mammalian cells, complement activation, and increased production of proinflammatory cytokines. In addition, the desired route of administration must be considered since it has been seen that these peptides can be easily degraded by proteases present in the blood or can bind to proteins, decreasing the desired effect, adding that under physiological conditions, some AMPs decrease or completely lose their bactericidal activity. This limitation, and others, such as the high production cost of these peptides, prevent AMPs from being used in clinical practice (127, 128).

## Discussion and future perspectives

It is accepted that AMPs exert a direct microbicidal effect and can cause changes in the host’s immune response, known as immune system modulation. These unique characteristics make them promising candidates for treating various infectious diseases. It is also known that bacterial infection control by these peptides is through their immunoregulatory functions. They can influence processes, such as immune cell chemotaxis, activation and differentiation of multiple cell lines, and the production of proinflammatory and anti-inflammatory cytokines. This type of modulation exerted by multiple AMPs represents an advantage in eliminating infections compared to the most commonly used antibiotics. The probability of resistant strains is reduced, in addition to their effect against existing multiresistant bacteria strains.

The use of AMPs or CPPs represents an even more successful therapeutic strategy in the context of infections caused by intracellular bacteria since several authors have confirmed that these peptides can cross eukaryotic membranes and exert a direct bactericidal effect on bacteria already inside the infected cell, with the bonus of the multiple regulatory effects exerted on the host’s immune system. Also, CPPs and AMPs can be conjugated with various types of molecules, including non-permeable antibiotics that favor successful interaction with intracellularly-located bacteria.

There are multiple reports on the bactericidal effect of various AMPs and CPPs on intracellular bacteria such as *Mycobacterium* and *Salmonella typhimurium*. It is important to emphasize that many of these studies have been *in vitro*, in a context where the bactericidal activity in the infected cell was not evaluated; therefore, this evaluation must be carried out in conditions that resemble the niche where this type of bacteria is usually found.

Although much research is lacking, there is no doubt that AMPs are a potential therapeutic agent in intracellular bacterial infections. Their mechanism of action encompasses various aspects, from the ability to directly kill bacteria to modulating the innate and acquired response of the host’s immune system and regulating inflammatory processes to control or eliminate the infection.

AMPs can be used as templates for drug development, preserving their immunomodulatory and bactericidal characteristics and trying to eliminate their adverse effects. Likewise, the new approaches using AMPs that include conjugation with different types of molecules increase the probability of success in eradicating intracellular bacterial infections.

## Author contributions

Both authors equally have made a substantial, direct, and intellectual contribution to the work and approved it for publication. 
